# Supplementary material for: ABCA1 Expression Is Upregulated in an EMT in Breast Cancer Cell Lines via MYC-Mediated De-Repression of Its Proximal Ebox Element
Source: Biomedicines. 2022 Mar 2;10(3):581. doi: 10.3390/biomedicines10030581 (PMC8945546; doi:10.3390/biomedicines10030581)
Supplement: Supplementary file 1 [file biomedicines-10-00581-s001.zip › biomedicines-1593244-supplementary.pdf]

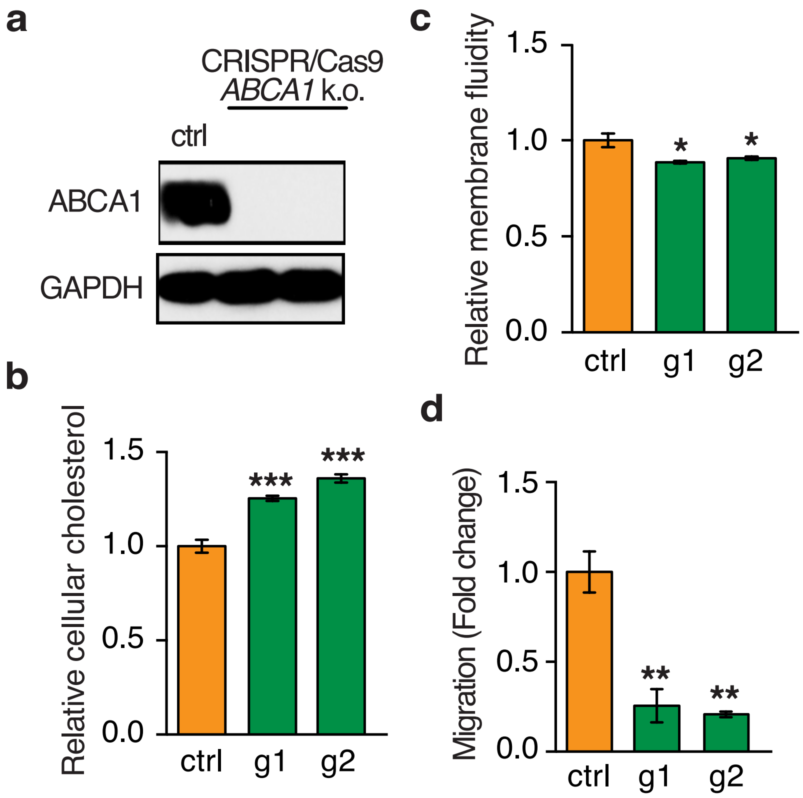

Figure S1. Knockout of ABCA1 results in reduced migration in metastatic breast cancer cells. (**a**) This immunoblot shows the expression of ABCA1 after CRISPR-mediated knockout (k.o.) using guide RNAs targeting either promoter 1 (g1) or exon 3 (g2). (**b**) The y-axis shows the cellular cholesterol levels of cells with and without ABCA1 knockout. (**c**) The y-axis shows the membrane fluidity of cells with and without ABCA1 knockout. (**d**) The y-axis shows the relative migration of cells with and without ABCA1 knockout. * $P<0.05$, ** $P<0.01$, *** $P<0.001$, **** $P<0.0001$.
